# Barriers to physical activity for father’s living in marginalising conditions

**DOI:** 10.1177/13634593211014275

**Published:** 2021-05-04

**Authors:** Francine E Darroch, John L Oliffe, Gabriela Gonzalez Montaner, Jessica M Webb

**Affiliations:** Carleton University, Canada; University of British Columbia, Canada; University of British Columbia, Canada; Independent Scholar, Canada

**Keywords:** gender and health, lifestyle, social inequalities in health

## Abstract

Physical activity can be a conduit for improving men’s social connectedness as well as physical gains for well-being. However, marginalised men, and fathers in particular, can be challenged to engage in leisure time physical activity. This qualitative study reports how fathers, who experience complex and significant social and health inequities, conceptualise and experience barriers to physical activity. Drawing from focus groups with 17 fathers, and semi-structured interviews with seven service providers about their perspectives on men’s physical activity in Vancouver’s Downtown Eastside (DTES), a highly marginalised neighbourhood. A masculinities framework was used to describe and contextualise physical activity in fathers’ lives. Three themes were inductively derived through the analyses: (1) ‘they’re busy surviving’ a finding referencing the work and limits invoked by poverty wherein survival was triaged ahead of leisure time physical activity; (2) ‘there is no activity centre’ chronicling the lack of physical activity spaces, programmes and resources available to fathers; and (3) ‘lifestyle affects our capability to exercise’ a theme detailing how social isolation amplified by factors including housing and opioid crises, and being a father in a resource poor setting imposed significant barriers to physical activity. The findings support reconceptualising physical activity programmes with men who are living in marginalising conditions to address behavioural and structural health inequities in tailoring father-centred programmes and resources.

## Background

Men’s reticence for self-health and health promotion programmes continues to be a prominent issue (Oliffe, 2015). Most often the estrangement of men from their health has been linked to their lower life expectancy wherein male morbidities and mortalities have been mapped to diseases and injuries amenable to prevention. For example, cardiovascular disease, suicide, motor vehicle accidents, infectious diseases (most often HIV) and liver failure account for Canadian men’s shorter lives compared to women (Goldenberg and Bilsker, 2011). Within the overarching category of men there are sub-groups who are especially challenged to promote their health and/or self-manage illness. Indigenous men, for example, have a much lower life expectancy compared to the general male population, and for Indigenous men who experience homelessness, poverty and substance use challenges, these inequities are significantly amplified (Kermode-Scott, 2005).

Within the broader context of men’s health promotion, running counter to many of the aforementioned challenges, are suggestions that men idealise physical activity because it aligns to masculine norms of competitiveness and performativity’s (Rørth et al., 2019). In addition, the numerous benefits of men’s physical activity are widely accepted and include improved psychosocial health, functional ability and overall quality of life (Warburton et al., 2006). Physical activity can also reduce men’s risk of coronary heart disease (Batty and Lee, 2004), depression (Teychenne et al., 2008) and dementia (Hamer and Chida, 2009). Physical activity is inclusive of all muscular movement with an increase in energy expenditure (World Health Organization [WHO], n.d.); leisure time physical activity is more precisely defined as behaviours consciously aimed at improving physical fitness (typically comprising sport, exercise, recreational walking) and not essential to activities for daily life (Moore et al., 2012). Leisure time physical activity is influenced by individual and sociocultural factors, and environments where people spend their time (Ross and Searle, 2019). Despite the widely accepted beneficial aspects of physical activity, inequitable access impacts who and how exercise is engaged (Hull et al., 2010; Rhodes et al., 2014; The Health and Social Care Information Centre, 2008). The literature points to various broad influences on men’s physical activity levels, including intra-personal, social and environmental factors (Sallis and Owen, 1999). Both income (The Health and Social Care Information Centre, 2008) and parenthood (Hull et al., 2010; Rhodes et al., 2014) are examples of factors known to impact men’s physical activity. Low income groups consistently show lower levels of physical activity (The Health and Social Care Information Centre, 2008). Further, chronic illnesses that can be offset with physical activity disproportionately affect low income males (Royal College of Physicians, 2004).

The transition to fatherhood is also associated with reduced physical activity (Hull et al., 2010; Rhodes et al., 2014) making fathers an important population for tailoring activity based health promotion programmes to. Fathers typically show high levels of inactivity due to barriers associated with inadequate time, guilt and low energy levels (Bellows-Riecken and Rhodes, 2008). In contrast, there is a positive association between economic resources and physical activity for men (Sharp et al., 2020). A US study reported that men in the highest income group had a 26% higher exercise energy expenditure and a 3% higher exercise intensity than those in the lowest income group (Meltzer and Jena, 2010). While leisure time physical activity is more common among affluent groups (Azevedo et al., 2007; Hallal et al., 2012) total physical activity including walking and other modes of active transport are inexpensive and accessible forms of physical activity for low income men (Monteiro et al., 2003).

There are significant gaps in the academic literature concerning physical activity for fathers who are highly marginalised (Hamilton and White, 2011); hence, little is known about the barriers and facilitators to achieve optimal wellness for these populations. Troublingly, fathers with young children are at greater risk of inactivity (Pot and Keizer, 2016). Further, Lee and Maheswaran (2011) noted that inner city and poor populations have lower levels of leisure time physical activity. Any form of physical activity is particularly important for these individuals, who may be at greater risk of anxiety, depression, low self-esteem and being overweight/obese (Evans et al., 2011). Fathers parenting in highly marginalising conditions are also at risk of being ineligible for some programmes and services – including physical activity interventions, by virtue of organisational policies (Darroch et al., in review). Research highlights the important influence fathers can have on children’s physical activity (Berge et al., 2010; Freeman et al., 2012; Wake et al., 2007); furthermore, there is reciprocity wherein physical activity participation can have positive effects on parenting practices (Hamilton and White, 2010).

In men’s health promotion there is significant evidence that exercise and activity-based programmes stand the best chance of meaningfully engaging end-users (Oliff et al., 2020a). As Connell (2005) suggested, masculinity is a verb – it is about doing – and physical activity can make available familiar terrain and performativities to many men (Oliffe et al., 2020b). Men’s wellness practices have also been associated with diverse enactments of masculinities that can negatively or positively impact men’s well-being (Oliffe et al., 2012a). Some men adapt masculine ideals by disregarding health promotion messages (Smith, 2007) and the way that men align, or not (and the continuum between those binaries), with masculine ideals can influence their health behaviours (Oliffe et al., 2012a). Indeed, fatherhood has been highlighted as a significant transition wherein men’s masculine norms can shift in response to the need for developing nurturing skills (Bottorff et al., 2010). It has also been recognised as a time where men may be motivated to change negative health practices (Bottorff et al., 2010; Williams, 2007). Researchers have argued that masculinities are a valuable frame for describing how gender specifically influences men’s wellness outcomes (Oliffe et al., 2012b; Sloan et al., 2010).

However, limited research has focused on the intersection of masculinity, fathers who are marginalised and resource poor and physical activity; as such, there is a gap in knowledge pertaining to the ways in which fathers prioritise physical activity with considerations of competing priorities. The current article addresses the research question; *What are the barriers to physical activity for father’s living in marginalising conditions?* to report how fathers, who experience complex and significant social and health inequities, conceptualise physical activity and experience barriers to physical activity.

## Methodology and methods

As part of a larger mixed methods project, data for the current study was collected May 2018 to May 2019 to better understand barriers to physical activity for marginalised fathers. Ethics approval was obtained from the University of British Columbia Behavioural Research Ethics committee. We conducted semi-structured interviews with service providers in Vancouver’s Downtown Eastside (DTES) and ran focus group interviews with fathers. The current article and analyses describe the barriers to physical activity for fathers in the DTES, thus we drew on data from two father focus groups (*n* = 17) (see Table 1) and key informant (service providers) (see Table 2) semi-structured individual interviews (*n* = 7). Men were eligible to take part in the study if they were aged 19 years or over, spoke English, lived in/or accessed services in the DTES and identified as a father. Eligible key informants were English speaking and worked in parent services providing sectors in the DTES. All participants were recruited through recruitment posters and snowball sampling.

**Table 1.** Participants.

Pseudonym	Age	Number of children currently in their care
Geoff	57	1
Robert	49	3
James	47	1
David	40	2
Greg	38	2
Peter	48	1
Colin	50	0
Steven	48	0
Sean	48	2
Roy	41	0
Ron	49	0
Frank	45	1
Kirk	44	0
Damien	56	1
Adam	39	0
Ray	41	0
Timothy	48	0

**Table 2.** Key informants.

Pseudonym	Sex	Years worked in the DTES
Sofia	Female	2
Kaitlin	Female	5
Lucia	Female	20
Michaela	Female	20
Caroline	Female	10
Luis	Male	5
Shannon	Female	11

This current study took place in the DTES, a relatively small geographic area with approximately 17,000 residents, many of whom experience precarious housing (City of Vancouver, 2011). While it is difficult to determine exactly how many people experience homelessness in the DTES, the Metro Vancouver 2017 homeless count reported 3605 people slept in shelters, tent cities or outdoors each night, 72% (1688) of whom identified as men (BC Non-Profit Housing Association and Thomson Consulting, 2017). This was a 30% increase from the 2014 homeless count. Resident’s experience high rates of substance use, communicable disease and mental illness compared to the general Vancouver population (Linden et al., 2013). The vast majority of the residents of the DTES are adults, and 38% of the residents live within family structures and 24% of these families are led by single parents with 19% of these single parent families being led by males (City of Vancouver, 2013).

There is also a need to contextualise the housing and opioid crises and their possible connections to physical activity for fathers in the DTES. In April, 2016 the province of British Columbia declared the opioid overdose crisis a public health emergency (City of Vancouver, n.d. [a]). Since the declaration, 1406 individuals have died across the province and Vancouver is the epicentre of this crisis (City of Vancouver, n.d. [a]). Provincial data revealed males accounted for 77% of all deaths (BC Coroners Report, 2019). Adding to this complexity, men are 55% more likely to meet the criteria for street homelessness and 85% of individuals who died as a result of the opioid crisis in 2016, were homeless men (BC Coroners Report, 2019). Over the past 10 years, as the opioid and housing crisis in Vancouver escalated, Oppenheimer Park, housed in the centre of the DTES, started to fill up with tents as people sought shelter. In September of 2019 there were over 130 tents set up in the small park (Vancouver Police, 2019). Historically, this park was a place of recreation and leisure, and the home field for the Asahi Japanese baseball team (Jette, 2007). The concerns and issues have been mounting with increased violent outbreaks which resulted in over 700 calls to police in 2019 about Oppenheimer park (Vancouver Police, 2019).

Participants in this study included fathers ranging in age from 38 to 58 years-old (mean 47 years), all of whom reported living with the aid of social assistance, 53% (*n* = 9) identified as Indigenous, 52% (*n* = 8) reported not currently providing direct care to their children, 59% (*n* = 10) reported spending one or more nights in a shelter in the past 12 months, with 18% reporting more than 15 days in a shelter in the past 12 months. Most men (77%; *n* = 13) reported ‘Yes’ to, *Do you think there is anything you can do to improve your physical health?* and 53% (*n* = 9) of the men reported that they currently exercised (see Table 3). The focus groups were co-facilitated by the first and fourth authors. All participants provided written informed consent prior to taking part and received a $25 honorarium, bus tickets and a meal during the focus groups. The questions guiding the focus group were semi-structured and included: *What challenges you most around doing physical activity? How does being a father impact your physical activity? What are some of the barriers to doing physical activity or accessing physical activity programmes in this neighbourhood? How might the challenges men face differ from women in accessing physical activity?* The focus groups lasted between 60 and 95 minutes.

**Table 3.** Demographics (*n* = 17).

Age (years)	
Range	38–58
Mean	46.94
Sexuality (%)	
Heterosexual/straight	82.4 (*n* = 14)
Gay or bisexual	–
You don’t have an option that suits me	17.6 (*n* = 3)
Indigenous identity (%)	
Yes	53 (*n* = 9)
No	47 (*n* = 8)
Education level (%)	
Some elementary school	5.9 (*n* = 1)
Elementary school	5.9 (*n* = 1)
Some secondary school/high school	35.3 (*n* = 6)
Completed secondary school/high school	17.6 (*n* = 3)
Some college or university	23.5 (*n* = 4)
Completed college or university	11.8 (*n* = 2)
Are you currently in a relationship with a partner? (%)	
Yes	23.5 (*n* = 4)
No	76.5 (*n* = 13)
In the last 12 months, have you spent one or more nights in any type of shelter? (%)	
Yes	59 (*n* = 10)
No	41.2 (*n* = 7)
How many nights in a shelter in the last 12 months? (%)	
1–7 nights	23.5 (*n* = 4)
8–14 nights	5.9 (*n* = 1)
15–30 nights	5.9 (*n* = 1)
More than 30 nights	11.8 (*n* = 2)
In general, how would you rate your overall health? (%)	
Excellent	–
Very good	5.9 (*n* = 1)
Good	35.3 (*n* = 6)
Fair	41.2 (*n* = 7)
Poor	11.8 (*n* = 2)
Are you currently being treated for depression, anxiety, post-traumatic stress disorder? (%)	
Yes	47 (*n* = 8)
No	47 (*n* = 8)
Don’t know	–
Experiences of discrimination in the past 3 months (%)	
Never	29.4 (*n* = 5)
Sometimes	47 (*n* = 8)
Often	17.6 (*n* = 3)

The key informants were also offered a $25 honorarium and the semi-structured interview questions included: *What are the barriers father’s face in accessing physical activity? How does your organisation’s policies support moms, dads, and both parents? How might these challenges be different and diverse for men and women?* The semi-structured interviews lasted 20–60 minutes; all were audio recorded, transcribed verbatim, accuracy checked and analysed to inductively derive thematic findings. Drawing on Connell’s (2005) masculinities framework our analysis is undertaken recognising dominant masculine ideals (e.g. provider, competitiveness, strength) and the plurality of masculinities embodied by men’s varied alignments to fathering and physical activity.

## Analysis

Thematic analysis was completed using Braun and Clarke’s (2006) six step approach. Data was uploaded to NVivo^10^. All authors read transcripts to ensure familiarity with the data and contexts under which it was collected. We then generated initial codes and assigned data segments to descriptive labels. In the next phase we discussed and examined the codes and organised and [re]assigned data to develop potential themes. We then reviewed and refined the preliminary themes to ensure they accurately represented the data and addressed our research question, *What are the barriers to physical activity for father’s living in marginalising conditions*. Next, we named and defined three themes reflecting the findings drawn from our analyses, or as Braun and Clarke (2006) describe we developed ‘the “essence” of what each theme is about. . .determining what aspect of the data each theme captures’ (p. 22). Finally, we wrote up the analysis drawing from the masculinities framework to elaborate and theorise the themes making linkages to existing empirical literature. Three themes were identified: (1) ‘they’re busy surviving’ a finding referencing the work and limits invoked by poverty wherein survival was triaged ahead of leisure time physical activity; (2) ‘there is no activity centre’ chronicling the lack of physical activity spaces, programmes and resources available to men; and (3) ‘lifestyle affects our capability to exercise’ a theme detailing how social isolation amplified by factors including the housing and opioid crisis and being a father in a resource poor setting imposed significant barriers to physical activity.

### Theme 1: ‘They’re busy surviving’

The first theme ‘they’re busy surviving’ referenced the limits and work invoked by poverty wherein surviving was triaged ahead of leisure time physical activity. As Michaela, a service provider with 20 years of experience suggested, ‘exercise in itself needs defining by the people who live here. What they see as exercise? Because people surviving or trying to deal with low amount of income or no income at all, they’re busy surviving’. Indeed, the fathers diversely explained physical activity as walking, jogging to pick-up goods, as well as central to games and team sports. Moreover, most of the physical activity they engaged was purposeful – comprising walking to and from locations to collect food or access social services. These errands were contrasted with, and prioritised ahead of, leisure time physical activity.

As Robert, a 49-year-old father explained, his physical activity was lifestyle related but indelibly linked to optimising his health;

For me it’s just being heathy. I do a lot of walking. That’s the extent of my exercise. And I walk lots, probably 2 to 5 miles a day, quite regularly, and for me it’s just I want to live longer. I want to be able to breathe better. I want to work on my internal body.

Robert, like many of the fathers, engaged in purposeful functional physical activity, walking everywhere and he didn’t consider leisure time physical activity as particularly important nor feasible in his life situation. Being healthy was important for Robert because it enabled him to complete his daily activities. Greg, a 38-year-old father shared his thoughts on physical activity but conceded that he could ‘do more’ leisure time activity;

I consider myself quite healthy and quite active, but I could do a lot more. [. . .] I’ve gotten a gym membership and never used it. That’s how a man thinks, so I quit buying them; I quit getting them. And there is a lot of activities that can be done here, but I don’t think I’m motivated enough to focus on my exercise. I would say I’m lazy or I’m a procrastinator.

Greg depicted a sensitivity to augmenting his physical activity with leisure time exercise in laying blame on his own poor work ethic as the major barrier to doing more. Indeed, many participants discussed individual behaviours ahead of citing their lack of capital and/or structural barriers such as cost and absent amenities. This might be understood as self-blame and buying into health promotion discourses that lobby ‘responsible’ citizens to do self-health.

That said, participants also frequently discussed complex DTES specific barriers to physical activity. In line with the above service provider’s comment, Frank, a 45-year-old father explained;

Some people around this area - they’re going from one place to eat to another place as exercise, because this is an all-day thing. Holy shit, it takes hours sometimes standing in line [. . .] That is almost a full-time job, seriously. It’s not because they’re forcing anyone to do it, it is just people are poor around here so it’s necessary. It is kind of hard to exercise when you’re doing shit like that and you’re trying to eat and you’re trying to get by, just trying get your next meal. It’s like I ain’t going to go exercise, it will burn energy and then I’ll have to go find food again. Shit, I’ll be hungry again.

This narrative illustrated the complex nature of physical activity for men living in poverty wherein being physically active required greater caloric intake. Men typically equated food as fuel, and in many examples shared by participants bulk was presumed to muster energy levels. By contrast, participant’s lack of access to food had implications for their energy and efforts towards conserving energy – and forgoing physical activity. Herein food insecurity posed a barrier to leisure time physical activity. Men had to plan their days around multiple organisation’s schedules in order to obtain food. As Michaela, a service provider with 20 years of experience working in the DTES explained;

Sometimes your mode of survival is really a lot of exercise, meaning you’re travelling up and down between East and West Hastings Street, which runs for [. . .] 3 km at least. So you are going up and down the street ten times a day to catch the food lineups so that you don’t have to be at the very back of the food lineup when the food runs out [. . .] People on average who go and do this urban hunting survival will tell you they have been walking all day nonstop.

In line with Frank’s explanation, Michaela referred to ‘urban hunting survival’ confirming the work demanded of fathers to overcome their lack of resources, and the challenges inherent to providing for family (and self). Lucia, a service provider with over 20 years of experience working in the DTES elaborated on this point;

They have so many other challenges and other obstacles that they’re facing on a daily basis. I think if we eliminated some of those such as housing, lack of food, daycare, they would shuffle their priorities and put exercise and their own wellbeing at the top, but the reality is it is not at the top because they face so many different challenges.

Caroline, a service provider of 10 years added that the timing of programmes and sometimes precarious nature of work schedules, did not support working fathers. She explained, ‘some of our dads work during the day or pick up labour work where they can, so it’s hard for them to access resources, daytime resources’. Of course, many of these challenges were highlighted in the context of fathering, and the layering of this role further complicated accessing leisure-time physical activity. Luis, who had worked in the DTES for 5 years with a fathers’ group explained;

There was a lot of pressure on men or dads to be the bread winner or be productive and active and assertive and all of those things, and when someone has experienced childhood trauma or mental health and addictions, [. . .] I think it makes it really hard to have a positive self-image and feel like you have, you’re a worthy person to exercise.

All of the fathers were economically disenfranchised, and the vast majority reported strained relationships with partners and/or their child(ren)’s mothers. The men detailed self and societal pressures to provide for their families and/or themselves in non-traditional jobs. In addition, it left some men time poor and/or self-assigning that they were undeserving of leisure-time physical activity, especially when it was a pursuit that might be judged as hedonistic. Participants also highlighted how patriarchal taxes can emerge for marginalised men, who despite lacking the masculine capital or privilege, expected (and strived for) to provide for family.

### Theme 2: ‘There is no activity centre here’

The second theme confirmed participant’s assertions that there was a lack of physical activity spaces, programmes and resources available to dads in the DTES. Most men agreed physical activity programmes were absent, as Peter, a 56-year-old participant explained;

There is no activity centre here. There is no place to go. A lot of the young people here are poor, especially the low-income teenagers [. . .], have nothing to do except drink. [. . .] But if there is a centre they can go to that they can be challenged in so many different ways, there’s coaches, there’s life coaches, and positive people there, they’ll do something. But here, it’s either you drink or you get high, so there is no place. . .there is no rec centre.

As Peter asserted, the scarcity of resources for men who were poor and living in the DTES was a barrier to physical activity. Moreover, he argued that substance use behaviours in the neighbourhood were fuelled by boredom and a lack of opportunities to address underlying traumas and issues. He also signalled that physical activity could be coupled with other services – in essence the development of holistic services to culturally norm physical activity as a gateway to wellness, personal growth and recovery. He suggested providing a facility that offered integrated tailored physical activity programming for DTES men.

Another participant, Adam, 39-years-old, added to this discussion stating, ‘I’ve lived in other provinces and they do offer these programmes, these leisure programmes, leisure passes, bus passes to get to these leisure programmes. BC (British Columbia), nothing, they don’t care’. The explicit barriers associated with cost were also highlighted, as Adam elaborated;

There is a lack of places to do it [physical activity], and even if you do there is always some sort of membership or something. There are all kinds of places, like there is the [name of fight club] over here and there is a couple of MMA [mixed martial arts] places you can go to a boxing club, but you need a membership and nobody has that kind of money.

In these examples, the structural barriers were highlighted as relative as well as overarching for men marginalised by poverty. The othering and oppression associated with the barriers to programmes that were appealing also permeated these men’s narratives. The net result was a lack of structure, and by extension a lack of routine that such physical activity could host, ultimately led men towards risky practices. Colin, a 50-year-old father, made a comment regarding barriers in the DTES;

If you go to a prison, there are activities to challenge us, challenge mentally. Here there is nothing, so getting in trouble, drinking, getting into fights, and especially summertime when people, everybody is always outside [. . .] Design a new rec centre in one of the abandoned buildings - design it for the people who live here and the challenges that they have.

Colin boldly suggested men from the DTES had more opportunities for addressing physical and mental challenges in prison in lobbying for tailored physical activity amenities. Within this context, the nature and specificities of the resource were referenced as critical to their effectiveness. In essence, the injustices of structural inequities were highlighted as barriers, in detailing the profound lack of opportunities for dads living in the DTES.

Service providers also offered explanations about the complexities of men accessing existing physical activity resources. For example, Sofia, with 4 years of experience in the DTES articulated the complicated relationships that some men had in accessing physical activity. She explained;

I think [. . .] confidence is a major factor. Going into new places or wellness-oriented spaces can be really intimidating for men as well [as women]. It’s easy for them to talk themselves out of it or feel like they don’t deserve to be there. They really need organizations and facilities to make them feel really welcome and comfortable in addition to addressing the barriers to access.

Sofia also noted the availability of physical activity programmes for men in the DTES, but recognised the exclusion of fathers with children in tow. She also conceded that organisations needed to do a better job in supporting fathers, and inclusively address broader determinants of physical activity if they were to improve leisure time physical activity. While service providers and fathers identified important barriers to accessing programmes and services in the community, Geoff, a 57-year-old participant argued there were ample programmes and services available. He stated;

Regarding programming, regarding activities, they’re everywhere. The question is how motivated are you, what are you willing to do. I mean we can sit here rationalizing forever and poor us [. . .] there is lots out there. There is community centres that have activities that you participate as a group [. . .] Memberships are only a dollar. Anybody can afford a dollar. I don’t really think there are any obstacles. I dealt with my alcohol and drug problem. That’s the underlying problem with our people down here, they’re addicts or they’re alcoholics, that’s the problem, and as long as they have that problem, they’re going to rationalize it forever. There are programs. It saddens the heart when I hear this – ‘oh we’ve got nothing, we don’t have a big gym, oh we don’t have this’. That’s bullshit, to me.

Geoff’s counter position called into question the other men’s grievances that physical activity facilities were absent or too costly to access. Interrogated were claims of hardship as well in pointing to the issues of substance use that were deemed to underpin so many men’s lack of self-health and reliance on social supports. This positioning might be understood as Geoff’s effort to jockey for position in lobbying *his* people to look inward – rather than outwardly for structural remedies to their challenges. Again, this position bought into the responsible citizen discourse that so often imbues men’s health promotion. While likely rendered as a call to action, Geoff also risked further marginalising other men who were profoundly and relatively without the capital or resources to take up his assertions and direction.

### Theme 3: ‘It’s kind of a lifestyle as well that affects our capability to exercise’

The third theme, ‘It’s kind of a lifestyle as well that affects our capability to exercise’ detailed how social isolation amplified by factors including the housing and opioid crisis, and being a father in a resource poor setting imposed significant barriers to physical activity. Within this context, social isolation emerged as a by-product of living with the scarcity that characterised the DTES and its men. Beyond surviving there was an underbelly of issues that prevailed to trump discussion about physical activity for fathers. There is recognition in the community that recreation sites are now being used as a strategy to address homelessness and an overall reflection of a lack of affordable housing in BC, and the DTES more specifically.

As Adam, a 39-year-old remarked;

The places that we have that are available for sports are being used for residents. People got their tents all over [. . .] It’s kind of a lifestyle as well that affects our capability to exercise, because you can’t really go play football when there are fucking tents everywhere, and when there are homeless people always right in your face. You can’t focus on what you’re doing because you’re feeling sad about why, what’s happening; it kind of takes that away from us. So every park you go to, you’re going to see it.

In examining place and space, disordered living was evident, simultaneously crowding and contesting the use of public environments in the DTES. Damien, a 56-year-old quizzically polled the other focus group participants, ‘Is it just me that finds it impossible to envision a game in a field with 100 tents on it? You can’t play baseball in a field full of tents’. While the dads were themselves precariously housed, their lifestyles were clearly impacted by the wider milieu and scarcity of housing – and by extension the lack of physical activity facilities in the DTES. Discussed were hierarchies of homelessness, with tent living posited as subordinate and negatively impacting all who lived in the DTES. Evident was the relative hardships and burdens that flowed to and from being marginalised. Reinforced was the struggle for position that comes within as well as across gendered hierarchies. In essence, some of the men asserted their distance from those living in tents to invoke the very ‘othering’ they themselves routinely lobbied against in their own lives.

The opioid crisis, similar to the tents, informed debates and divisiveness wherein the dominant DTES cultures contrasted buyers and sellers, drug users and recovering men to the point where structure and agency imbalances and their role in overdose deaths was entirely absent. Distrust drove stoicism and distance for many fathers wherein staying clean took precedent ahead of any endeavours for physical activity. For example, Steven, casually mentioned he valued the fathers’ group he was attending because it provided ‘a bit of stability, some place that you can go to once a week and be normal instead of out there grinding all week’. The grind of the lifestyle was hard both mentally and physically on the men, and they were ever vulnerable to giving in to the pressures. These vulnerabilities were also typically muted, though the safe places available within the dads group meetings permitted and affirmed men’s talk of such uncertainties and doubt. Steven’s comments, although seemingly contradictory, illustrated the plurality and transformative potential of men’s identities. His experience was common among the participants, and substantiated by service provider, Luis who explained;

It was a group designed to focus on supporting fathers in the community, but it is also kind of a men’s wellness space as well, some of the guys coming to the group don’t have access to their kids or might just be seeing them once of week kind of thing. A lot of the group is kind of about the positive identity of being a father and just guys in the community supporting each other [. . .] Generally it’s just, it will be just day to day life stuff or guys living in the neighbourhood and lots of stuff around mental health and addictions and kind of those challenges and housing. Then there are a few that will be connecting with their kids regularly and that’s a space for them to talk about that as well.

As both Steven and Luis suggested, many of the men felt isolated, and despite the male hierarchies within and outside of the DTES community, there was a desire to connect and trust. The group Luis ran provided an opportunity for men to connect informally for coffee and food. Luis further pushed for opportunities for the men to be physically active, to address something he saw as lacking in the community. As Kaitlin, another service provider who had worked with both mothers and fathers for 5 years in the DTES noted;

Dads are even less acknowledged than moms in the community- it is really hard to even figure out where to start with them. Some are single dads and need childcare, some don’t see their kids, some are supporting moms however they can [. . .] within this neighborhood, dads are just much more isolated.

Kaitlin’s comments confirmed the isolation fathers could experience, and the challenges of defining and delivering tailored services them. Michaela, a service provider further explained;

What I’m saying is, we need to have a balance of psychosocial activities and a convening safe environment that is conducive to community development and cohesion. I believe this aspect of a physical [. . .] it is about human connections. That’s a big part of it, in addition to our health and physical wellness.

Michaela stressed the importance of community and human connection as critical to improving overall well-being in the DTES community, and particularly for fathers. Creating opportunities for social connection through shared activities, such as physical activity/sport, may be an important unifying strategy moving forward, not only for individuals but for transforming masculine norms at the community level. In line with Michaela’s commentary, Shannon, a service provider stated;

I would like to see more programming for the dads and some of the boys in the building. I’d like to see some good opportunities for good modeling. I think when the dads here participate more it creates more of a, just a stronger community fabric. To see men who live here doing things to support the building and to support the community, makes an impact on people.

Shannon shared her desire for programming that was geared towards fathers as she identified a need for opportunities to model healthy behaviours and potentially shift the masculine norms in the DTES context. Herein the potential to nurture and build transformative masculine norms were positioned as remedy – both in terms of addressing the divisiveness of the housing and opioid crises – as well as building a collective of fathers with shared values for connecting and confiding.

## Discussion

While physical activity has been espoused as the gateway to successful men’s health promotion programming (Bottorff et al., 2015; Sharp et al., 2020c) the current study findings provide important cautions and caveats to those widely held beliefs. Likewise, the fathering constructs embodied by most participants revealed distinct challenges and practices that clearly resided outside dominant ‘involved fathering’ discourses (Pot and Keizer, 2016). Indeed, ever clear were the layering effects of men’s health inequities, and the importance of working with those limits (and the men’s strengths) to advance their health – and by extension the health of their families. The current work contributes to a small but important body of work focussed on men’s physical activity with an emphasis on fathers who experience multiple marginalities. Our findings align with but also elaborate on this work – especially research with men who are often labelled as ‘hard to reach (and research)’ (Oliffe et al., 2017; Bottorff et al., 2015; Lefkowich et al., 2015). Key findings point to context specific considerations to address social isolation among fathers in the DTES, and the importance of creating opportunities for fathers to connect through shared activities. As Oliffe et al., (2017) noted, men connect through activities, and shared roles (fathering) and/or challenges (to provide for self and family). However, programme pacing and the proportion of physical activity content need to facilitate opportunities for men to connect within group-based programmes. Within this context, we argue that the typical DTES lifestyle and dominant cultures need to be transformed within programmes to permit and affirm connectedness among fathers (as a gateway to *some* physical activity). While physical activity can be a draw, and social connection an outcome of fathers’ gatherings – it might be that in the specific context of marginalised fathers we do not need the ‘prop’ of physical activity. Said another way, that the barriers to physical activity, as detailed in our findings, are so great, there is likely significant benefit to offering a dads group – and threading some physical activity to those gatherings. Herein men’s health inequities should take precedent in tailoring programmes for fathers in the DTES. Ever clear are the shortcomings of assuming men’s unitary alignments to physical activity. Rather, the centrality of recognising multiple masculinities in targeting specific end-user sub-groups with community-based programmes is deeply evident. Contrasting physical activity-based programmes such as Hat Trick (Caperchione et al., 2016) who purposefully attracted overweight and obese middle aged men and the Football Fans in Training programme (Hunt et al., 2014) who catered to Scottish men challenged by low income and job insecurity, it is clear that fathers in the DTES were unlikely to identify with sports teams let alone travel to those arena venues to take part. In what follows we discuss each of the thematic findings to further develop these key points.

The first theme richly described the work and limits invoked by poverty wherein men’s survival was triaged ahead of leisure time physical activity. Prioritising leisure time physical activity was challenging for the men, both in terms of competing priorities and their purpose driven physical activity (i.e. gathering food, accessing services). Drawing from Connell’s (2005) masculinities framework this prioritising of DTES fathers might be reasonably argued as reflecting alignments to provider and protector roles. Thoughtfully considering these contextual findings, Lefkowich et al.’s (2015) assertion resonated in that gender specific strategies were clearly needed to engage DTES men. That might usefully include acknowledgment (and affirmation) of what fathers already do in terms of their physical activity in the day to day, and offering programmes that value add to men’s survival work. As Oliffe (2015) suggested physical activity programmes have to be tailored to male sub-groups to engage them with health promotion.

When considering health promotion strategies for fathers in the DTES, framing an intervention as something *other than* health benefits may also be an effective strategy. For example, the review by Brown et al. (2016) highlighted the *Healthy Dads Healthy Kids’* intervention pointing out how deliberately marketing opportunities to spend quality time with children, rather than a strategy to improve men’s health outcomes (e.g. weight loss) drew strong participation from fathers. The men who took part in our research also relayed that physical activity for the sake of advancing one’s health was not a priority. Therefore, a tailored ‘intervention’ might profit by trading on the masculine ideals of doing health for the benefit of others. For example, a fitness or conditioning level to make available performance-based efficiencies that dads can use in their work of securing resources for their families.

The second theme chronicled the lack of physical activity spaces, programmes and resources available to men. Although we are not questioning the experiences of the men, there is a need to clarify contrasting viewpoints in theme two. There are numerous programmes, activities and services provided to men living in the DTES (although not necessarily fathers). In fact, Vancouver, like many other cities across Canada, runs a leisure access programme which provides low income individuals and families with ‘basic recreation programs and services’ (City of Vancouver, n.d. [b]). Only two of the participants in our study had knowledge of this programme. It is clear there is a need to increase awareness of the programmes available to fathers in the DTES. The limited knowledge of existing leisure access programmes and other community centre services is problematic. Summers et al. (2004) likewise noted low income fathers are often unaware of services, thus unable to find them or unable to prioritise. The vast majority of the fathers in our work were unlikely to prioritise these ‘services’ or to be actively looking for such ‘non-essential’ programmes. The fathers were likely blinded to these opportunities in their efforts to secure basic necessities. A key learning from this finding is that ‘new’ and existing programmes need to be visible but also in line with the men’s life challenges. Where our findings differ from those commonly cited in the literature, is that labelling physical activity programmes, as such, may not be the most effective strategy to engage men in the DTES. Moreover, incentivising fathers to participate in physical activity programmes might rely on providing food to more aptly situate men’s connectedness as a value-added variation to their day-to-day lives.

There must also be considerations for the naming and mode of delivery of physical activity – to promote and ensure success of programmes and the potential to promote men’s health. We recommend a move away from siloed approaches to health and social support by incorporating physical activity to some mutual help groups that men regularly access in the DTES. More importantly, we suggest renewed consideration of the broader structural issues associated with being precariously housed, experiencing economic inequalities, and the impact of discrimination on fathers in the DTES (Darroch et al., in review). Such commitments take the onus off men’s health behaviours to integrate efforts towards increased funding for longer-term support services to integrate physical activity.

The third theme detailed how social isolation was entwined with place specific housing and opioid crises, and how being a father in a resource poor setting invoked significant barriers to physical activity. Many of the fathers were estranged or had limited access to their children. The discussions reflected a marginalised masculinity and a gender hierarchy which rendered some men subordinate within that milieu. This might be understood as competing masculinities wherein fathers devoid of masculine capital (i.e. absence of involved fathering, secure work and housing) pointed to their struggles and resilience to differentiate their work rate from other lesser men. Within this context, although we were interested to better understand participants fathering practices and perspectives, the focus group participants collectively worked around what were likely sites of shame and conflict. Research conducted by Bunn et al. (2016) suggested health promotion activities to support lifestyle changes may be more effective if there are shared experiences and cultural commonalities among participants. In line with these strategies, future programming and resources for fathers in the DTES might incorporate group work that affirms lived experiences amid seeking to transform (and transgress) some gendered norms to make available social connectedness and therapeutic conversations that unmute men’s stoicism. These additional supports, coupled with physical activity may yield positive mental, physical and social health outcomes for fathers, and their children.

Locations must also be conducive for facilitating men’s physical activity engagement, therefore partnering with trusted community groups who work with men or fathers in the DTES is key. As Oliffe et al., (2020b) recommended, ‘gender-sensitized, purpose-built men’s health promotion programmes, based on understandings of the intended end-user’s race, culture, socioeconomic status, education and income levels, and the intersections with masculine roles, relations and identities gave rise to important tailored programs’ (p. 2). At the time of this research, half of the fathers reported a limited parental role, and there may be value in creating programming specifically for this sub-population to provide further contextualised support. Service providers in the DTES are an extremely valuable source of information and should be key partners in reimagining, co-creating, launching and facilitating services for, and with, fathers.

The current study limitations provide direction for future research. First, our small sample size may not be representative of the experiences of fathers in the DTES. Expanding the current research to increase the sample size and gain additional insights, specifically with fathers who are not actively parenting, would be helpful in addressing this limitation. Second, while we examined multiple marginalities among the men, there was limited discussion of culture. A large proportion of the participants in this study were Indigenous men, and though gendered patterns were distilled, further examining the intersections of masculinity and men’s Indigenous identities in the context of fatherhood and physical activity might advance the field. Third, while we found the fathers experienced multiple barriers to leisure time physical activity, they appeared to be quite physically active. Despite the participants in this study reporting little to no engagement in leisure time physical activity, future research might usefully examine how much lifestyle physical activity (e.g. walking) men living in marginalising conditions complete. Based on focus group data, many of the participants suggested they may far exceed daily recommendations based on Canadian Physical Activity Guidelines (Canadian Society for Exercise Physiology, n.d.). Notwithstanding the aforementioned limitations, this research contributes crucial empirical insights about fathers living in marginalising conditions and their barriers to leisure time physical activity.

## Conclusion

There is a dearth of research focused on physical activity for fathers living in marginalising conditions. This current research captures some challenges for resource poor fathers in thoughtfully considering the ways physical activity might be further integrated into their lives and marginalising conditions. Central are men’s health inequities and the complexities that accompany those marginalised yet resilient masculinities. Importantly, there are opportunities to re-imagine physical activity in the DTES context. Oliffe et al., (2012a) argue for strength-based approaches and messaging privileging the experiences of fathers to foster connections between masculine ideals and self-health. It is clear that DTES cultures need to be understood in terms of what counts most as strengths amongst fathers. To this point – we might inadvertently ostracise fathers by being blind to their current physical activity while naming leisure time physical activity as a programme focus.

Creating opportunities for fathers to connect in ways that align, and ideally transform some of their masculine ideals, might provide opportunities for social connection and fathering practices that men are more comfortable talking about. In order to be responsive to men’s health inequities, we recommend re-framing and gender sensitising men’s leisure time physical activity to recognise the potential of physical activity as a background programme part, the potential for reducing social isolation through father gatherings, and the ever-present need to address structural barriers disproportionality assigned to marginalised men.
